# Supplementary material for: Identification of Chemical Vapor Mixture Assisted by Artificially Extended Database for Environmental Monitoring
Source: Sensors (Basel). 2022 Feb 3;22(3):1169. doi: 10.3390/s22031169 (PMC8840270; doi:10.3390/s22031169)
Supplement: Supplementary file 1 [file sensors-22-01169-s001.zip › Supporting Information.pdf]

Supporting Information for

Identification of chemical vapor mixture assisted by artificially extended database for environmental monitoring

Hi Gyu Moon[1,†],Youngmo Jung[2,†], Beomju Shin[2], Donggeun Lee[2], Kayoung Kim[2], Deok Ha Woo[2], Seok Lee[2], Sooyeon Kim[1], Chong-Yun Kang[3,4], Taikjin Lee[2,*] and Chulki Kim[2,*]

[1] Center for Ecological Risk Assessment, Korea Institute of Toxicology (KIT), Jinju 52834, Republic of Korea

[2] Sensor System Research Center, Korea Institute of Science and Technology (KIST), Seoul 02792, Republic of Korea.

[3] Center for Electronic Materials, Korea Institute of Science and Technology (KIST), Seoul 136-791, Republic of Korea.

[4] KU-KIST Graduate School of Converging Science and Technology, Korea University, Seoul 02841, Republic of Korea.

*e-mail:taikjin.lee@kist.re.kr, chulki.kim@kist.re.kr

[†] They made an equal contribution to this work

Table of Content

Table S1. Identification accuracy of individual vapors and mixture vapors

Figure S1. Concept of data processing model to classify the mixture vapors based on database of individual vapors using by pattern recognition.

Figure S2. Experimental response curve of metal oxide sensor array for (a-b) NO_2, (c-d) NH_3, and (e-f) HCHO vapors in 150 and 200°C.

Figure S3. Experimental response curve of metal oxide sensor array for (a-b) NO_2 + NH_3, (c-d) HCHO + NH_3, (e-f) NO_2 + HCHO and (g-h) HCHO + NO_2 + NH_3 vapors in 150 and 200°C.

Figure S4. Two dimensional PCA results of mixture vapors (NO_2 +HCHO, NO_2 + NH_3, HCHO + NH_3, and HCHO + NO_2 + NH_3), which are projected to PC plane of individual vapors. In the inset area, all of vapors can be well classified.

Table S1. Identification accuracy of individual vapors and mixture vapors

Type	Vapors	Test	Results	Accuracy
Individual	NO_2	9	9	100%
	NH_3	9	9	100%
	HCHO	9	9	100%
Mixture	NO_2+NH_3	9	9	100%
	NH_3+HCHO	9	8	89%
	HCHO+NO_2	9	8	89%
	NO_2+NH_3+HCHO	9	8	89%

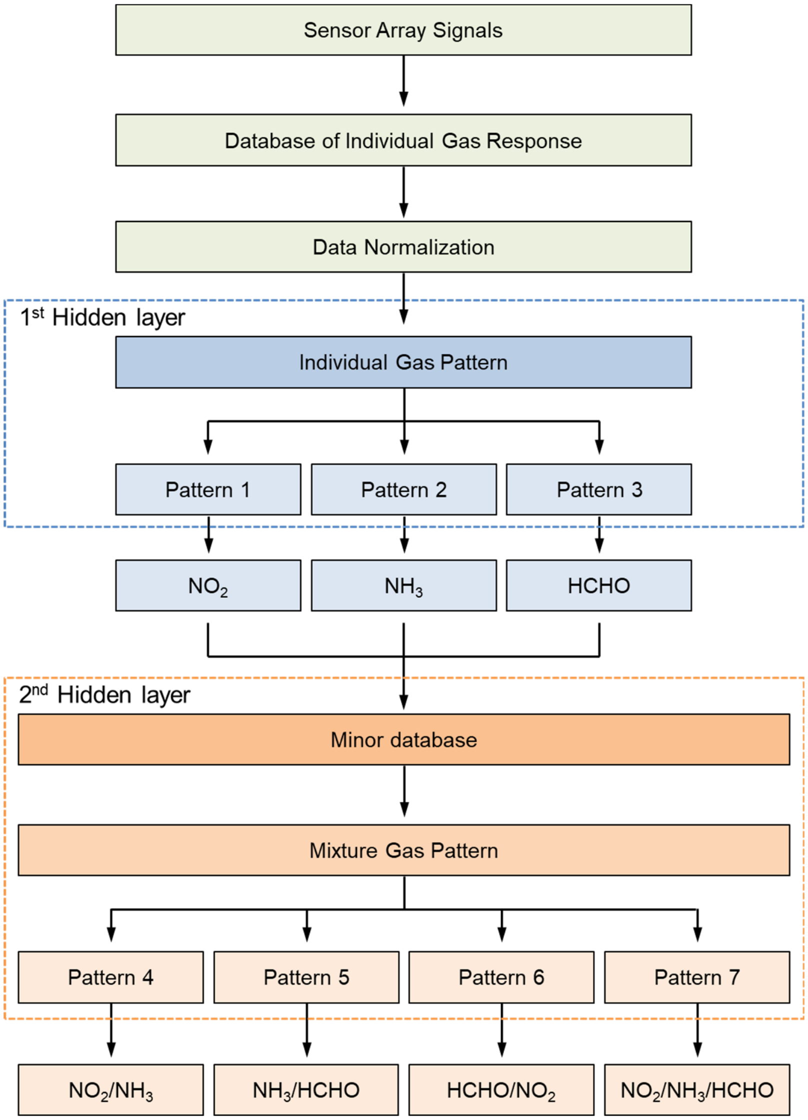

Figure S1. Concept of data processing model to classify the mixture vapors based on database of individual vapors using by pattern recognition.

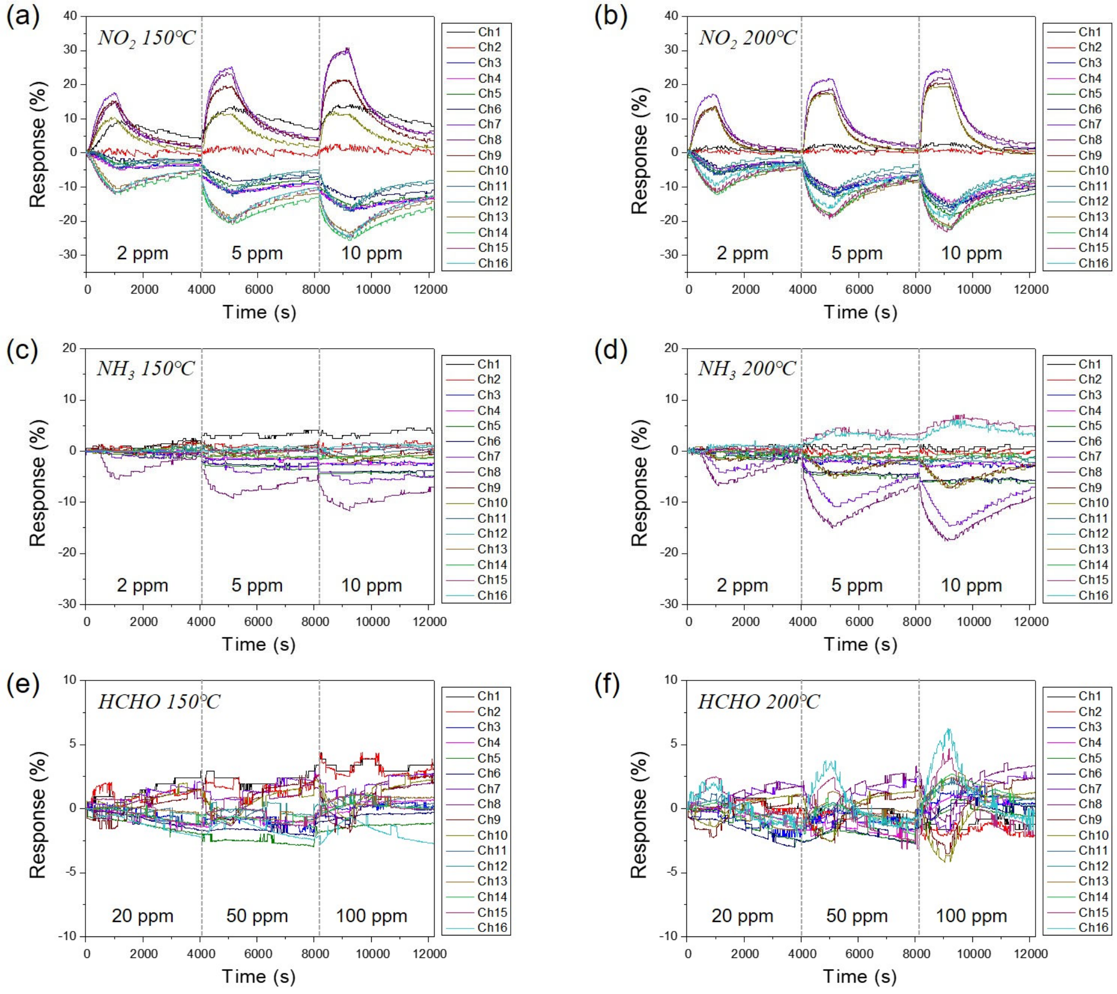

Figure S2. Experimental response curve of metal oxide sensor array for (a-b) NO_2, (c-d) NH_3, and (e-f) HCHO vapors in 150 and 200℃.

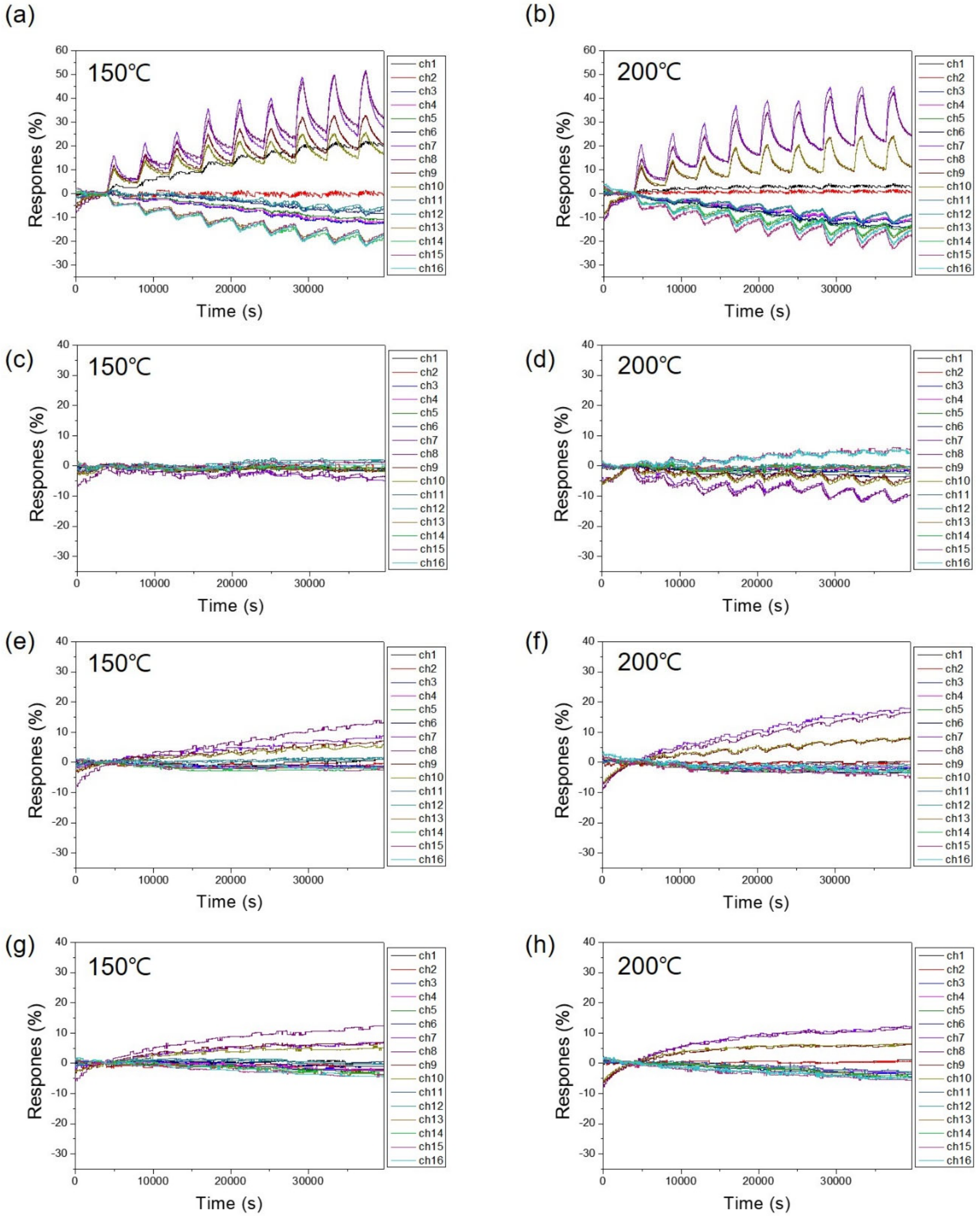

Figure S3. Experimental response curve of metal oxide sensor array for (a-b) NO_2 + NH_3, (c-d) HCHO + NH_3, (e-f) NO_2 + HCHO and (g-h) HCHO + NO_2 + NH_3 vapors in 150 and 200℃.

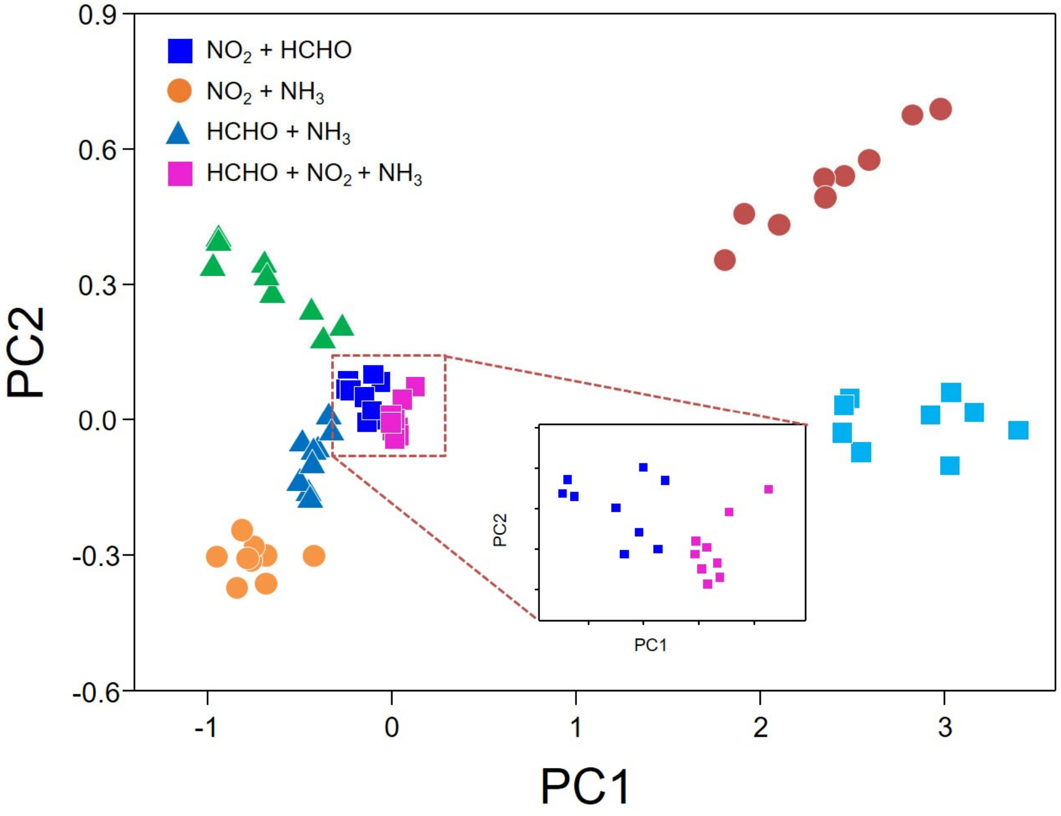

Figure S4. Two dimensional PCA results of mixture vapors (NO_2+HCHO, NO_2 + NH_3, HCHO + NH_3, and HCHO + NO_2 + NH_3), which are projected to PC plane of individual vapors. In the inset area, all of vapors can be well classified.
